# Jasmonic acid biosynthesis by fungi: derivatives, first evidence on biochemical pathways and culture conditions for production

**DOI:** 10.7717/peerj.10873

**Published:** 2021-02-05

**Authors:** Felipe Eng, Jorge Erick Marin, Krzysztof Zienkiewicz, Mariano Gutiérrez-Rojas, Ernesto Favela-Torres, Ivo Feussner

**Affiliations:** 1 Department of Plant Biochemistry, Albrecht-von-Haller-Institute for Plant Sciences, University of Goettingen, Goettingen, Germany; 2 Biotechnology Division, Cuban Research Institute on Sugar Cane Byproducts (ICIDCA), Havana, Cuba; 3 Laboratório de Processos Biológicos, Escola de Engenharia de São Carlos, Universidade de São Paulo (LPB/EESC/USP), São Carlos, Brasil; 4 Campus Iztapalapa, Biotechnology Department, Universidad Autónoma Metropolitana, Mexico City, Mexico; 5 Department of Plant Biochemistry, Goettingen Center for Molecular Biosciences (GZMB), University of Goettingen, Goettingen, Germany; 6 Department of Plant Biochemistry, International Center for advanced Studies of Energy Conversion (ICASEC), University of Goettingen, Goettingen, Germany

**Keywords:** Oxylipin, Fungi, Submerged fermentation, Jasmonic acid, Culture medium, Metabolic pathway

## Abstract

Jasmonic acid (JA) and its derivatives called jasmonates (JAs) are lipid-derived signalling molecules that are produced by plants and certain fungi. Beside this function, JAs have a great variety of applications in flavours and fragrances production. In addition, they may have a high potential in agriculture. JAs protect plants against infections. Although there is much information on the biosynthesis and function of JA concerning plants, knowledge on these aspects is still scarce for fungi. Taking into account the practical importance of JAs, the objective of this review is to summarize knowledge on the occurrence of JAs from fungal culture media, their biosynthetic pathways and the culture conditions for optimal JA production as an alternative source for the production of these valuable metabolites.

## Introduction

Jasmonic acid (JA) and its derivatives belong to a group of plant hormones called jasmonates (JAs) (Wasternack & Feussner, 2018). They belong to the large group of oxidized lipid signalling molecules, so-called oxylipins (Gerwick, Moghaddam & Hamberg, 1991). In plants, JAs derive either from α-linolenic acid (18:3(n-3)) or raughanic acid (16:3(n-3)) and their major representatives are the isomers (+)-7-*iso*-JA and (−)-JA. These compounds are widely distributed in algae (Ueda et al., 1991), angiosperms (Wasternack & Hause, 2013) and certain fungi (Hause et al., 2007; Miersch et al., 1993). They belong to the group of phytohormones playing a role as growth inhibitors and regulating plants defence responses (Pieterse et al., 2009; Wasternack et al., 2006).

Methyl jasmonate (MeJA) was firstly isolated as an odoriferous constituent of the essential oil of *Jasminun grandiflorum* and other plant species (Crabalona, 1967; Demole, Lederer & Mercier, 1962). It is recognized as an important ingredient in high-grade perfumes, cosmetics and in the preparation of detergents, soaps and food aromas with floral notes (Asamitsu et al., 2006; Dhandhukia & Thakkar, 2007a). JA was first isolated as plant growth inhibitor from cultures of the fungus *La*s*iodiplodia theobromae* (synonym *Botryodiplodia theobromae*) (Aldridge et al., 1971).

JA and MeJA have attracted the attention of plant physiologists since the development of efficient methods for detecting and quantifying metabolites about 35 years ago. The presence of these compounds in different parts of plants was initially correlated with their strong promotion of senescence and inhibition of growth in angiosperms when applied exogenously (Wasternack & Hause, 2002). Although these compounds act as growth inhibitors or senescence promoters at high concentration, they induce the expression of defensive genes at much lower levels. For instance, they promote the synthesis of proteinase inhibitors, enzymes of phytoalexin synthesis, thionins, defensins and the vegetative storage protein genes (Howe & Jander, 2008).

However, JAs play an important role in agriculture nowadays by regulating the defence of plants against pests and pathogens (Gális et al., 2009; Gavin et al., 2012; Hawkins et al., 2007; Heil et al., 2001; Moreira et al., 2019; Rohwer & Erwin, 2008; Sanches et al., 2017; Stout, Zehnder & Baur, 2002; Wasternack, 2014). Their application seems to be in line with the principles of sustainable agriculture since they may be less aggressive to the environment than pesticides and mineral fertilizers (Secatto, 2013).

Furthermore, it has been observed that adding exogenous of MeJA stimulates the production of many secondary metabolites in cell suspension cultures, such as taxane and derivatives from *Taxus* sp (Yukimune et al., 1996) and camptothecin production from *Ophiorrhiza mungos* L. (Deepthi & Satheeshkumar, 2017). These metabolites are very promising anticancer drugs in humans (Miller, Neilan & Sze, 2008; Sriram et al., 2005). Although credible evidence on a mechanism of action was missing until recently (Bömer et al., 2020). Studies have been conducted to optimize the production of these substances; focusing on their metabolic pathways, selecting more productive cell lines, optimizing cell culture processes, product purification, and up scaling of the whole process (Bai et al., 2004; Miller, Neilan & Sze, 2008; Onrubia et al., 2013; Syklowska-Baranek et al., 2009; Tabata, 2006; Wilson & Roberts, 2012).

Currently most of the aroma compounds including JAs may be extracted from natural plant sources. However, recent advances in metabolic engineering have generated a great interest to produce these substances from alternative sources (Gupta, Prakash & Gupta, 2015). An alternative and attractive route for producing JAs could be based on microbial biosynthesis and biotransformation. Microorganisms such as bacteria and yeast can be used at variable scales as safe producers of flavours and fragrances (Gill & Valivety, 1997). Most importantly, these microorganisms can be metabolically and genetically modified to enhance the production of the desired metabolites. Moreover, the production of aroma compounds from microbial cultures or their enzyme preparations offers several advantages over traditional methods. The microbial metabolites can be produced in large quantities by using a fermentation process and can give high yields in very good qualities with better product characteristics along with low economical costs (Gupta, Prakash & Gupta, 2015).

Presently there are numerous projects ongoing for sequencing the genomes of ascomycete fungi (http://mycocosm.jgi.doe.gov/pages/fungi-1000-projects.jsf) and one of them is dealing with the JAs producing fungus *L. theobromae*. From this project, valuable information will be available in the near future that will help to continue the analysis of fungal JA biosynthesis and other related metabolites using a reverse genetic approach. In fact, the lasiodiplodin biosynthetic gene cluster from the genome of *L. theobromae* strain NBRC 3,1059 was expressed in *Saccharomyces cerevisiae* strain BJ5464 to obtain a phytotoxic polyketide that inhibited human blood coagulation factor XIIIa, mineral corticoid receptors and prostaglandin biosynthesis (Xu et al., 2014).

## Survey Methodology

Scientific reports and patents dealing with the production and properties of JAs are still steadily increasing (Pirbalouti, Sajjadi & Parang, 2014; Wasternack, 2015). However, there are few reports related to the production of JAs by fungi. Therefore, the aim of this review is to discuss the existing reports related to the fungal production of JAs focusing on the type of fungus, biosynthetic pathways, and culture conditions. By screening the publicly available databases Free Patents Online (http://www.freepatentsonline.com/), Google Patents (https://patents.google.com/), Espacent (https://worldwide.espacenet.com/), Google Scholar (https://scholar.google.de/), PubMed (https://www.ncbi.nlm.nih.gov/) and Web of Science (https://apps.webofknowledge.com/), we aimed to cover the current status of the field and apologize to scientists whose work we overlooked.

## JAs from Fungi

**Figure 1:**
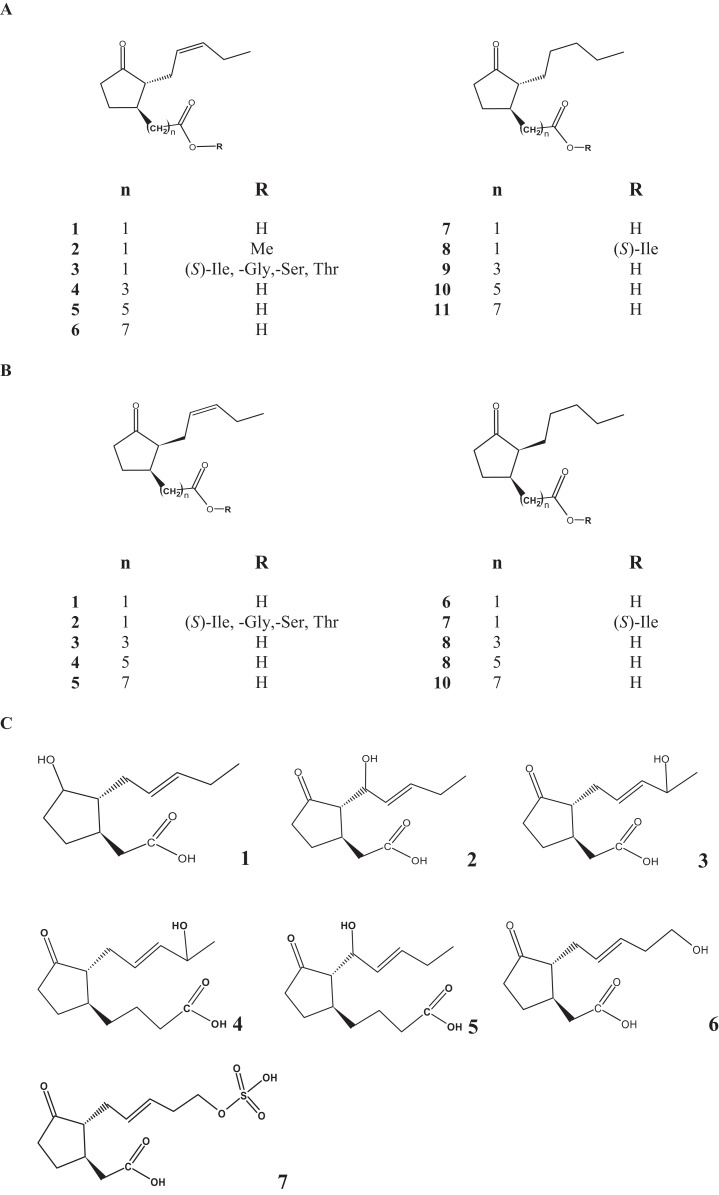
Chemical structure of the most important jasmonates found in fungi. (A) *Trans*-compounds: 1, jasmonic acid; 2, jasmonic acid methyl ester; 3, jasmonoyl isoleucine, glycine, serine and threonine conjugates; 4, 3-oxo-2-(2-pentenyl)-1-butyric acid; 5, 3-oxo-2-(2-pentenyl)cyclopentane-1-hexanoic acid; 6, 3-oxo-2-(2-pentenyl)cyclopentane-1-octanoic acid; 7, 9,10-didehydro-JA; 8, 9,10-dihydro-7-iso-jasmonoyl-isoleucine; 9, 3-oxo-2-pentanylcyclopentane-1-butyric acid; 10, 3-oxo-2-pentanylcyclopentane-1-hexanoic acid; 11, 3-oxo-2-pentanyl)cyclopentane-1-octanoic acid. (B) *cis*-compounds: 1, jasmonic acid; 2, jasmonoyl isoleucine, glycine, serine and threonine conjugates; 3, 3-oxo-2-(2-pentenyl)-1-butyric acid; 4, 3-oxo-2-(2-pentenyl)cyclopentane-1-hexanoic acid; 5, 3-oxo-2-(2-pentenyl)cyclopentane-1-octanoic acid; 6, 9,10-didehydro-JA; 7, 9,10-dihydro-7-iso-jasmonoyl-isoleucine; 8, 3-oxo-2-pentanylcyclopentane-1-butyric acid; 9, 3-oxo-2-pentanylcyclopentane-1-hexanoic acid; 10, 3-oxo-2-pentanyl)cyclopentane-1-octanoic acid (all of them was found with trans- or cis-attached side chains). (C) 1, 2-(-3-hydroxy-2-(-pent-2-en-1-yl)cyclopentyl)acetic acid; 2, 8-hydroxy jasmonic acid; 3, 2-(-2-(-4-hydroxypent-2-en-1-yl)-3-oxocyclopentyl)acetic acid; 4, 4-(2-(4-hydroxypent-2-en-1-yl)-3-oxocyclopentyl)butanoic acid; 5, 4-(-2-(-1-hydroxypent-2-en-1-yl)-3-oxocyclopentyl) butanoic acid; 6: tuberonic acid; 7: 12-hydroxy jasmonic acid sulfate.

*Lasiodiplodia theobromae* is a common phytopathogenic fungus capable of producing JAs at high level as a result of its primary and secondary metabolism (Alves et al., 2008; Eng et al., 2016; Salvatore, Alves & Andolfi, 2020). Although, JA is produced as the main product, other JAs such as 9,10-didehydro JA (9,10-ddh-JA), 11-hydroxy JA and 12-hydroxy JA sulfate (12-HSO_4_-JA) were formed to a lesser extent (Fig. 1; Table 1) (Eng, 2012; Miersch et al., 1987). Cucurbic acid (CA) that may also be recognized as a phytohormone and synthesized by a so far unknown pathway has been also detected in trace amounts (Eng, 2012; Miersch et al., 1987).

**Table 1:** Occurrence of jasmonic acid and other jasmonates from plants and microorganisms.

Jasmonates	Plant	Fungi
Jasmonoyl isoleucine, glycine, serine, threonine, phenylalanine, tyrosine, tryptophan, leucine, isoleucine conjugates	Hamberg & Gardner (1992); Svoboda & Boland (2010)	Cross & Webster (1970); Miersch et al. (1992); Miersch, Bohlmann & Wasternack (1999); Castillo et al. (2014); Cole et al. (2014), Oliw & Hamberg (2017), Oliw & Hamberg (2019)
9,10-didehydro-JA	Hamberg & Gardner (1992)	Eng (2012), Oliw & Hamberg (2019)
9,10-dihydro-7-iso-jasmonoyl-isoleucine	Sembdner, Atzorn & Schneider (1994)	Cross & Webster (1970); Miersch et al. (1992); Miersch, Bohlmann & Wasternack (1999), Oliw & Hamberg (2019)
3-oxo-2-(2-pentenyl)cyclopentane-1-butyric acid, 3-oxo-2-(2-pentenyl)cyclopentane-1-hexanoic acid, 3-oxo-2-(2-pentenyl)cyclopentane-1-octanoic acid	–	Miersch, Bohlmann & Wasternack (1999)
Curcurbic acid	Sembdner & Parthier (1993)	Miersch et al. (1987); Eng (2012)
8-hydroxy-jasmonic acid	Hamberg & Gardner (1992)	Miersch, Schneider & Sembdner (1991)
11-hydoxy-jasmonic acid	Wasternack (2006)	Miersch, Schneider & Sembdner (1991)
12-hydoxy-jasmonic acid or tuberonic acid	Hamberg & Gardner (1992); Wasternack (2006)	Miersch, Schneider & Sembdner (1991)
12-hydoxy-jasmonic acid lactone, tuberonic acid-O-β-glucopyranoside, curcubic acid-O-β-glucopyranoside	Hamberg & Gardner (1992)	–
3-oxo-2(1-hydroxy-2’-pentenyl)-cyclopentane-1-butanoic acid, 3-oxo-2(4-hydroxy-2’-pentenyl)-ciclopentane-1-butanoic acid	–	Miersch, Schneider & Sembdner (1991)
12-hydroxy jasmonic acid sulfate	Gidda et al. (2003)	Eng (2012)
4,5 didehydro-7-isojasmonic acid, 3,7-didehydrojasmonic acid, 6-epi-curcubic acid lactone, Homo-7-isojasmonic acid, Dihomo-7-isojasmonic acid, 11-hydroxi-dihomojasmonic acid, 8-hydoxy-dihomojasmonic acid	Hamberg & Gardner (1992); Asamitsu et al. (2006)	–
Methyljasmonate	Seo et al. (2001); Cheong & Choi (2003)	Andolfi et al. (2014)
*cis*-Jasmone	Steinegger & Hansel (1988); Koch, Bandemer & Boland (1997)	Matsui et al. (2017)

Overall 8 hydroxy JAs (11-hydroxy JA, 12-hydroxy JA or tuberonic acid (TA), 8-hydroxy JA, 3-oxo-2(1-hydroxy-2′-pentenyl)-cyclopentane-1-butanoic acid and 3-oxo-2(4-hydroxy-2′-pentenyl)-cyclopentane-1-butanoic acid) were detected in the culture medium and biomass of *L. theobromae* strain D7/2 growing in a medium containing sucrose, soy flour, corn steep liquor and a mineral salt solution (Miersch, Schneider & Sembdner, 1991). Twenty-two JAs were identified after 8 weeks of culture of *Fusarium oxysporum* f sp *matthiole* strain 247.61 grown in liquid potato-dextrose broth under static conditions (Miersch, Bohlmann & Wasternack, 1999). Among the metabolites produced, 9,10-dihydro-7-*iso*-jasmonoyl-isoleucine, jasmonoyl-isoleucine (JA-Ile), 9,10-dihydro jasmonoyl-isoleucine, 3-oxo-2-(2-pentenyl)cyclopentane-1-butyric acid, 3-oxo-2-(2-pentenyl)cyclopentane-1-hexanoic acid and 3-oxo-2-pentylcyclopentane-1-octanoic acid were identified. The isoleucine conjugates were also produced by the culture of *Gibberella fujikuroi* (Miersch et al., 1992). Interestingly, *F. oxysporum* f sp *mattiole* was unable to accumulate any hydroxylated-JAs as shown for *L. theobromae* (Miersch et al., 1993).

The occurrence of the JA-serine and JA-threonine conjugates was confirmed in the fermentation broth from *Lasiodiplodia* sp. strain 2,334 using HPLC-ESI tandem mass spectrometry in negative ionization mode, while JA-glycine and JA-isoleucine conjugates were identified with the same technique but with positive ionization (Castillo et al., 2014). In higher plants, JA amino conjugates are regular constituents accumulating upon sorbitol treatment or wounding (Guranowski et al., 2007; Miersch, Bohlmann & Wasternack, 1999).

While the conjugating enzyme was first isolated form the flowering plant *Arabidopsis thaliana* (Staswick, Tiryaki & Rowe, 2002), the corresponding peptidase activity was isolated from *L. theobromae* strain D 7/2 (Hertel et al., 1997). This enzyme was capable of hydrolysing JA-conjugates with α-amino acids. The enzyme was purified by gel filtration, ion exchange and hydrophobic interaction chromatography. It was characterized as glycoprotein with a molecular mass of about 107 kDa and its amidohydrolase activity was very specific with regard to (−)-JA and α-amino acids with (*S*)-configuration. Therefore, the authors suggested that this fungus may need this enzyme during infection of the host plant for start or modify plant processes, for example, senescence or the release of nutrients, which probably being beneficial for the fungal growth.

JA, MeJA and three JA esters, named lasiojasmonates (botryosphaerilactone A, (3*S*,4*R*,5*R*)-4-hydroxymethyl-3,5-dimethyldihydro-2-furanone and (3*R*,4*S*)-botryodiplodin) were detected from culture filtrates of *Lasiodiplodia* sp. strain BL101 isolated from declining grapevine plants that showed wedge-shaped cankers (Andolfi et al., 2014). However, phytotoxic assays recording necrotic lesions on grapevine and cork oak leaves demonstrated that only JA was found to be active.

The diversity of octadecanoid and jasmonoyl compounds found in the culture filtrate of these fungi raise the question whether the compounds are formed only or at least primarily during the interaction with plants and, if so, what the function of these compounds might be. Evidence suggests that fungal pathogens exploit host oxylipins to facilitate their development via inducing plant lipid metabolism to utilize plant oxylipins in order to promote G-protein-mediated regulation of sporulation and mycotoxin production in the fungus and use of host-ligand mimicry to manipulate plant defence responses from which the fungus benefits (Christensen & Kolomiets, 2011). However, in others cases *F. oxysporum* colonization remains symptomless or even has beneficial effects on plant growth and/or stress tolerance. Moreover, in pathogenic interactions, a lengthy asymptomatic phase usually precedes disease development. All this suggests for a sophisticated and fine-tuned interaction between *F. oxysporum* and its host (Di, Takken & Tintor, 2016).

Phytotoxic metabolites were identified in the culture media of six species of *Lasiodiplodia* isolated in Brazil causing *Botryosphaeria* dieback of grapevine (Cimmino et al., 2017). It was found by LC-MS, that only four of these strains (*L. brasiliense, L. crassispora, L. jatrophicola* and *L. pseudotheobromae*) produced JA. *L. brasiliense* also synthesized also (3*R*,4*S*)-4-hydroxymellein. This was the first report on JA production from these species. Fungal-derived *cis*-jasmone (CJ) was detected in *L. theobromae* strain MAFF 306027 (Matsui et al., 2017). These authors carried out studies of the deuterium labelled metabolism of 18:3(n-3)-*d*_5_, OPC:4-*d*_6_, OPC:6-*d*_6_, OPC:8-*d*_6_ and *cis*-OPDA-*d*_5_ to MeJA-*d*_5_ and/or CJ-*d*_5_ in feeding experiments with this strain, revealing that the fungus produced CJ through a single biosynthetic pathway via *iso*-12-oxo-phytodienoic acid (*iso*-OPDA). Interestingly, it was suggested that the previously predicted decarboxylation step of 3,7-didehydro JA to afford CJ might be not involved in CJ biosynthesis in *L. theobromae* (Matsui et al., 2017). However, in plants CJ is synthetized from 18:3(n-3) via two biosynthetic pathways using JA and *iso*-OPDA as key intermediates (Koch, Bandemer & Boland, 1997).

In spite of the diversity of JAs produced by fungi, JA is the metabolite that has aroused the most interest, due to a higher concentration detected in the culture media, the variety of their applications and their high market values. A large number of fungi JAs with similarities to the JAs of plants could show that probably the biosynthetic pathways and the intermediates involved in fungi and plants are similar.

## JA Biosynthetic Pathway

### Plants

Many reviews have summarized the developments on the biosynthetic pathway of JA in plants and our knowledge will be briefly summarized in the following section (Agrawal et al., 2004; Creelman & Mullet, 1997; Goepfert & Poirier, 2007; Hamberg & Gardner, 1992; Schaller, Schaller & Stintzi, 2005; Vick & Zimmerman, 1984; Wasternack & Feussner, 2018; Wasternack & Hause, 2002; Wasternack & Hause, 2013).

**Figure 2:**
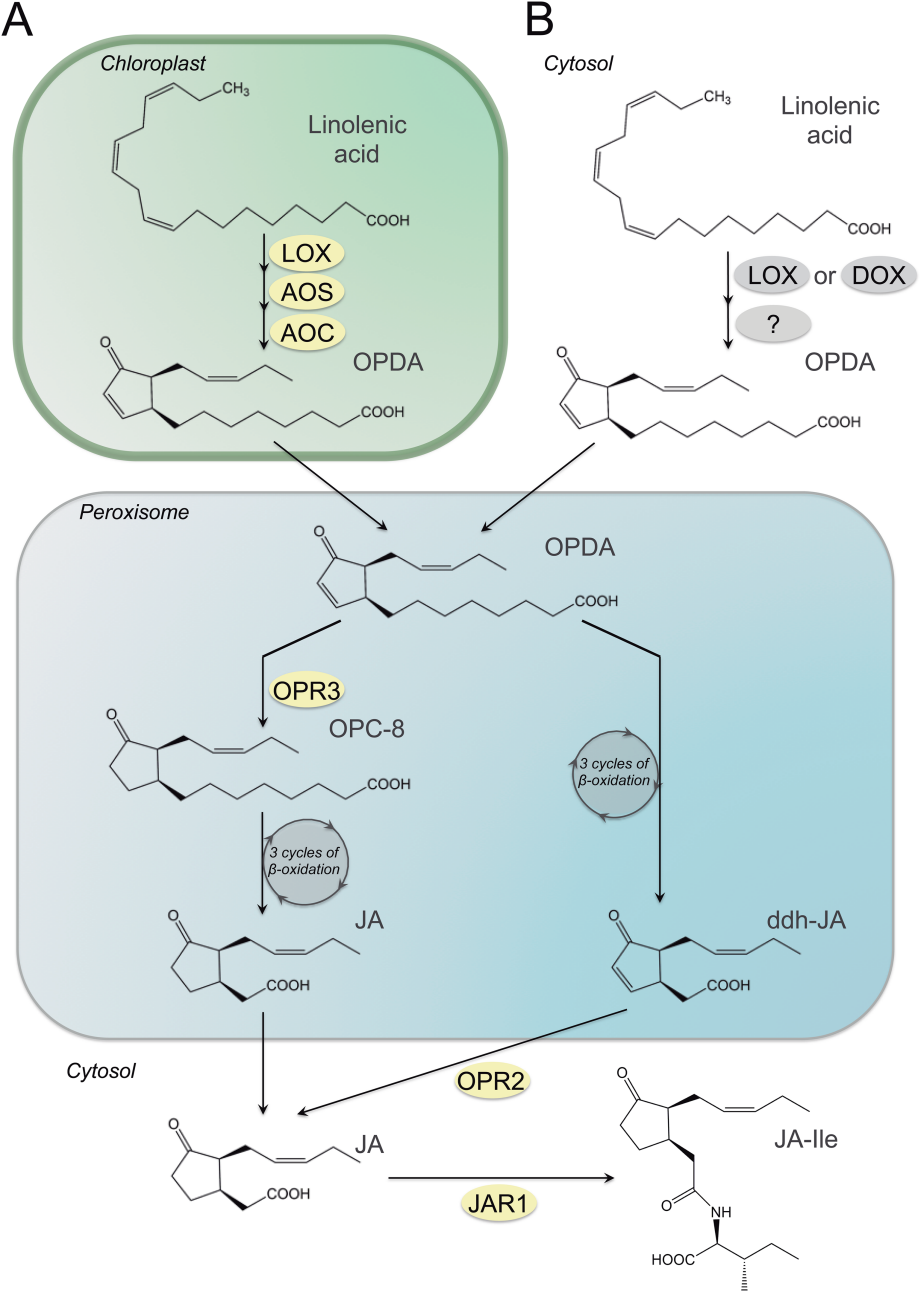
Synthesis of JA and its amino acid-conjugate JA-Ile in plants and fungi. Synthesis of JA and its amino acid-conjugate JA-Ile in plants (A) and a scheme for OPDA formation in fungi. (B) Enzymes known only for *Arabidopsis thaliana* are indicated in yellow circles and those known from fungi are marked by grey circles. Abbreviations: AOC, allene oxide cyclase; AOS, allene oxide synthase; ddh-JA, 4,5-didehydro jasmonic acid; JA, jasmonic acid; JA-Ile, jasmonic acid isoleucine conjugate; JAR1, jasmonoyl amino acid conjugate synthase; LOX, lipoxygenase; OPC:8, 3-oxo-2-(2-pentenyl)-cyclopentane-1-octanoic acid; OPDA, cis-(+)-12-oxo-phytodienoic acid; OPR2,3, 12-oxo-phytodienoic acid reductase.

JA biosynthesis in plants starts with the liberation of 18:3(n-3) or 16:3(n-3) from the plastid envelope membranes by lipases (shown in Fig. 2 for 18:3(n-3)). This reaction as well as the next three steps of the pathway are localized in plastid ending with the formation of either *cis*-(*+*)-12-oxo-phytodienoic acid (OPDA) or *dinor*-oxo-phytodienoic acid (*dn*-OPDA), respectively. This is the result of the sequential action of the enzyme lipoxygenase (LOX), allene oxide synthase (AOS) and allene oxide cyclase (AOC) on 18:3(n-3) or 16:3(n-3). The next steps take place in peroxisomes where OPDA and *dn*-OPDA are activated and reduced to 10,11-dihydro-12-oxo-phytodienoic acid (OPC-8) and 3-oxo-2(2′-pentenyl)-cyclopentane-1-hexanoic acid (OPC-6) by 12-oxo-phytodienoate reductase isoenzyme 3 (OPR3), respectively. These reactions are followed by two or three rounds of β-oxidation, yielding OPC-6; 3-oxo-2(2′-pentenyl)-cyclopentane-1-butanoic acid (OPC-4) and finally (+)-7-*iso*-JA that rearranges into the (−)-JA isomer (with an molar ratio of 9:1 for (−)-JA/(+)-7-*iso*-JA) (Wasternack & Hause, 2013). JA can be further metabolized into its methyl ester (MeJA) by JA carboxyl methyltransferase (JMT) (Cheong & Choi, 2003; Seo et al., 2001), or by conjugation with amino acids (such as leucine and isoleucine) or sugars, respectively (Haroth et al., 2019; Oliw & Hamberg, 2019; Sembdner & Parthier, 1993; Wasternack, 2016).

Recently an alternative pathway was discovered in *Arabidopsis thaliana* via direct β-oxidation of OPDA leading to formation of 4,5-ddh-JA which is then reduced by OPR2 to JA (Chini et al., 2018). Whether the reactions downstream from OPDA may follow preferentially this pathway or the OPR3-dependent pathway needs to be answered by future research (Han, 2017) (Fig. 2).

### Fungi

Knowledge about metabolic pathways leading to the production of JAs by fungi is scarce (Han, 2017). Therefore, more physiological and biochemical studies are required and the existing data will be summarized throughout the next paragraphs.

Starting with the products formed, the same ratio of isomers (−)-JA:(+)-7-*iso*-JA that was found in plants was measured in the culture filtrate of *F. oxysporum* strain 247.61 (Miersch, Bohlmann & Wasternack, 1999). By contrast, only the (+)-7-*iso*-JA isomer was found in a culture of *L. theobromae* strain D7/2 (Miersch et al., 1987), but later both isomers, with a ratio of ~15:1 and 1:1 in two different experiments in the culture medium filtrate from *Lasiodiplodia* sp. strain 2,334, were described (Jernerén et al., 2012).

For *L. theobromae*, it was shown that JA production derived from 18:3(n-3) by using a culture medium that was supplemented either with ^13^C-sodium acetate or (^2^H_6_)-18:3(n-3) (Tsukada, Takahashi & Nabeta, 2010). Appreciable amounts of (^13^C)-JA and (^2^H_5_)-JA were detected in the culture supernatants, and the MeJA of OPDA was detected in mycelium extracts.

The studies in the JAs biosynthesis from *F. oxysporum f*. sp. *tulipae* led also the detection of some key intermediates involved in this pathway (Oliw & Hamberg, 2017; Oliw & Hamberg, 2019). This strain released over 230 mg L^−1^ of (+)-JA-Ile and with about 10 times less 9,10-dihydro-(+)-7-*iso*-JA-Ile from potato dextrose broth cultures when shaking at 100 rpm and 28 °C after 15 days. Incubation of mycelium of *F. oxysporum f*. sp. *tulipae* with radiolabelled *d*_5_-18:3(n-3) was able to detected an allene oxide and 12-OPDA derivative. They concluded that allene oxide was formed by a cytochrome P450 or catalase-related hydroperoxidase and 12-OPDA by an allene oxide cyclase (AOC) and, as the plants; this fungus forms JAs with an allene oxide and 12-OPDA as intermediates (Oliw & Hamberg, 2017). However, incubation of mycelia from this fungus with radiolabelled *d*_5_-18:2(n-6) was oxidized to 12-oxo-10-phytoenoic acid and finally converted to 9,10-dihydro-JA-Ile in analogy with the reactions of 18:3(n-3) via 12-OPDA into JA-Ile in fungi and plants by LOX, AOS and AOC. Therefore, the fungal AOC has a broader substrate specificity than the plant AOC, but it may form these intermediates from allene oxides by the same reactions (Oliw & Hamberg, 2019). Then, these results suggest that JA is synthesised by these strains from 18:3(n-3) via OPDA and that the enzymes involved may be similar to those governing JA biosynthesis in higher plants. However, there are probably also some differences in the genes and enzymes of the JA pathway between plants and fungi. For example, although higher plants and the fungus *G. fujikuroi* produce structurally identical gibberellins (GAs) using similar steps, there are important differences in pathways and enzymes involved (Hedden, 2008). These profound differences suggest that higher plants and fungi have evolved their complex biosynthetic pathways to GAs independently and not by horizontal gene transfer.

In fact, the fatty acid composition in *Lasiodiplodia* sp. strain 2,334 showed that the mycelium contained polyunsaturated C18 fatty acids, including 18:3(n-3) as probable substrate for JA biosynthesis (Eng, 2012; Eng et al., 2016; Jernerén et al., 2012). However, polyunsaturated C16 fatty acids were not detected (Jernerén et al., 2012). OPDA and OPC:4 were also detected in culture filtrates from this fungus as probable intermediates on the JA pathway (Eng, 2012; Eng et al., 2016). In addition, the JA precursors 3-oxo-2-pentylcyclopentane-1-butyric acid, 3-oxo-2-(2-pentenyl)cyclopentane-1-hexanoic and 3-oxo-2-(2-pentenyl)cyclopentane-1-octanoic acid were detected in a culture filtrate from *F. oxysporum* f sp *matthiole* strain 247.61 (Miersch, Bohlmann & Wasternack, 1999; Miersch et al., 1993, 1989). These data suggest that an OPR3-dependent JA biosynthesis pathway exists in this fungus (Fig. 2, left peroxisomal pathway).

Studies aiming at identifying single steps in fungal JA biosynthesis have been reported using different exogenously applied substrates (Jernerén et al., 2012), a reverse genetic approach (Brodhun et al., 2013) and enzyme purification (Patel, Patel & Thakkar, 2015). In the first case, a fatty acid dioxygenase activity from three strains of *Lasiodiplodia* was described (Jernerén et al., 2012). Two of the strains revealed low secretion of JA (~0.2 mg L^−1^). These strains oxygenated 18:3(n-3) to 5,8-dihydroxy linolenic acid as well as to 9*R*-hydroperoxy linolenic acid, which was further metabolized by an AOS activity into 9-hydroxy-10-oxo-12*Z*,15*Z*-octadecadienoic acid. Analogous conversions were observed with linoleic acid (18:2(n-6)) as a substrate. Studies using (11*S*-^2^H)18:2 revealed that the putative 9*R*-dioxygenase catalysed the stereospecific removal of the 11*R* hydrogen followed by a suprafacial attack of dioxygen at C-9. Mycelia from these strains contained 18:2 as the major polyunsaturated fatty acid but lacked 18:3(n-3). The third strain however secreted high amounts of JA (~200 mg L^−1^). It contained 18:3(n-3) as major fatty acid and produced 5,8-dihydroxy linolenic acid from exogenously added 18:3(n-3). From these three strains together no enzyme activity pointing to a JA pathway and being similar to that of higher plants could be identified.

As no sequence information on the *L. theobromae* genome is yet available, a reverse genetic strategy focused on a 13-LOX from *F. oxysporum* that may initiate JA production was used as second approach. It was based on using sequences similar to those found from enzymes being part of the JA biosynthetic pathway of plants (Brodhun et al., 2013). One of the sequences called FoxLOX was cloned and expressed in *E*. *coli*. FoxLOX was found to be the only non-heme Fe-LOX in the genome, which oxidizes polyunsatured C18 fatty acids to 13*S*-hydroperoxy derivatives by an antarafacial reaction mechanism where the *bis*-allylic hydrogen abstraction is the rate-limiting step. Having 18:3(n-3) as substrate, FoxLOX was found to exhibit a multifunctional activity, because the hydroperoxy derivatives formed were further converted to dihydroxy-, keto- and epoxy alcohol derivatives. The identification of FoxLOX as a specific linoleate 13*S*-LOX could suggest a JA biosynthetic pathway in *F. oxysporum*, which is analogous to that in plants.

A LOX enzyme was purified from the mycelium of *L. theobromae* strain MTCC 3,068 by chromatography (Patel, Patel & Thakkar, 2015). It was found that this fungus contains two LOXs isoenzymes, one of 93 kDa (LOX1) and another of 45 kDa (LOX2). The latter being most likely a degradation product of LOX1. Both LOX isozymes oxidized linoleic acid to produce a mixture of 9- and 13-hydroperoxy linoleic acid, optimum pH at 6 and temperature in the range of 40–50 °C. Therefore, both or one these LOX may be another candidate enzyme involved in fungal JA production.

It could be expected that fungal LOX involved in JA pathway contains Fe or Mn as the catalytic metal, whereas animals, plants and prokaryotes express LOX with only Fe (Wennman et al., 2016). Moreover, fungal LOX could have Ile- or the Val-group in the amino acid sequence. The Ile-group has a conserved WRYAK sequence that appears to be characteristics for these enzymes and has a C-terminal amino acid Ile. While the Val-group has a highly conserved WL-L/F-AK sequence that is also found in LOXs of plant and animal origin. Some LOXs have signal sequences implying these LOXs will be expressed extracellular (Heshof et al., 2014).

Therefore, these first data suggest, that JA may be synthesised from 18:3(n-3) via OPDA in fungi (Fig. 2) as was mentioned previously. Since fungi do not have plastids, the reactions leading to the formation of OPDA most likely take place in the cytosol or associated to a membrane leaflet facing the cytosol by LOX, AOS and AOC (Oliw & Hamberg, 2017). Whether this pathway may be initiated by LOX enzymes or other dioxygenases (DOX) is still unclear, just like the identity of the following enzymatic activities. However, the reactions downstream from OPDA may follow a discovered pathway in *Arabidopsis thaliana* via direct β-oxidation of OPDA leading to formation of 4,5-ddh-JA, which could be reduced by a fungal OPR2 homologue to JA (Chini et al., 2018) or/and the OPR3-dependent pathway needs to be addressed by future research (Han, 2017) (Fig. 2).

## Chemical Synthesis of JAs

Chemical synthesis and isolation of JAs from microorganisms and plants started in the 70s of the last century (Aldridge et al., 1971). JA is traditionally isolated from plants; mainly from jasmine and tea flowers. A large number of flowers produce small amounts of essential oils. For instance, it takes about 500 Kg of petals to obtain approximately 1 Kg of rose oil and this is a very expensive and time-consuming process that accounts for the high price of these oils (Dhandhukia & Thakkar, 2007a). Therefore, this is a very expensive and time-consuming process that accounts for the high price of these oils (Dhandhukia & Thakkar, 2007b). Consequently, numerous and different chemical synthesis strategies for obtaining JA, MeJA and other derivatives have been developed. In that way, some of these strategies are summarized below. The synthesis of MeJA and methyl curcubate (MeCA) have been reported by using 2-allylcyclohexan-1,3-dione as starting compound and hydroboration-oxidation followed of seven or eight steps for the first and second product, respectively (Kitahara et al., 1987). Moreover, the same authors improved the total yield for MeJA to up to 20% in twelve reaction steps by improving the stereoselectivity of the hydroboration-oxidation by using 3-hydroxy methylcyclopentanone as starting compound (Kitahara et al., 1991).

Shortly after these reports, racemic 7-substituted derivatives of MeJA have been synthesized (Taapken et al., 1994). 7-Methyl MeJA was also synthesized in enantiomerically pure form in seven steps from the Hajos-Wichert ketone. In addition, the biological activity of the prepared compounds has been investigated for the induction of tendril coiling in *Bryonia dioica* and the elicitation of the phytoalexin production in *Eschscholtzia californica*. However, beside 7-methyl MeJA all synthesized compounds showed poor activity in the bioassays (Taapken et al., 1994).

An interesting methodology leading only to racemic MeJA was proposed by Dos Santos et al. (2001) based on the synthon equivalent to a carboxymethyl anion to enones and nitroalkenes, through a 1,4-addition reaction of 2,4,4-trimethyl-2-oxazoline cyanocuprate 3; afforded the (±)-MeJA in 32% overall yield from 2-cyclopenten-1-one.

Suzuki, Inomata & Endo (2004) developed a new method of MeJA and MeCA synthesis using a chiral tricyclic lactone as starting compound via a new type of tandem retro-Diels-Alder-ene reaction activated by a trimethysilyl substituent as the key step, followed at seven reaction steps.

Other authors have dedicated their efforts to the synthesis of β-oxidation intermediates of JA, such as 10,11-dihydro-12-OPDA (OPC:8) and *cis*-(+)-OPDA by chemical or enzymatic means with good yields (Löwe, Dietz & Gröger, 2020; Nonaka et al., 2010; Takayuki, Michitaka & Yuichi, 2003; Zerbe, Weiler & Schaller, 2007).

JA and TA (Fig. 1C, free fatty acid is shown as compound 6) were synthesized from the key aldehyde, all *cis*-2-(2-hydroxy-5-vinylcyclopentyl)acetaldehyde, which was in turn prepared stereoselectively from the (1*R*)-acetate of 4-cyclopentene-1,3-diol through a S_N_2-type allylic substitution with CH_2_-CHMgBr followed by Mitsunobu inversion, Eschenmoser–Claisen rearrangement, and regioselective Swern oxidation of the corresponding bis-TES ether. A Wittig reaction of the aldehyde with (PH_3_P(CH_2_)Me)^+^Br^-^ followed by oxidation afforded JA stereoselectivity over the *trans* isomer (Nonaka et al., 2010). Similarly, TA was synthesized.

Secatto proposed a racemic synthesis of JA involving additional steps to obtain higher yield. This would envisage an application at industrial scale (Secatto, 2013). This synthetic route consisted of seven steps with an overall yield of 30%. The improvement of this route is due to the use of a starting compound without hygroscopic characteristics and no requirement for any pretreatment and easy handling. Moreover, the starting materials (adipic acid and cyclohexane and ethanol as solvents) are not expensive, leading overall to low production costs.

Two macrolactones (JA-Ile-lactones) derived from 12-OH-JA-Ile were synthesized in seven steps with an overall yield of 33% from commercially available MeJA (Jimenez-Aleman et al., 2015a). The biological activity of macrolactones was tested for their ability to elicit nicotine production, a well-known jasmonate dependent secondary metabolite. Both macrolactones showed biological activity, inducing nicotine accumulation to a similar extent as MeJA does in *Nicotiana attenuata* leaves. Surprisingly, the highest nicotine contents were found in plants treated with the JA-Ile-lactone, which has (3*S*,7*S*) configuration at the cyclopentanone ring and is yet not known from natural JAs.

A new synthetic route to JA-Ile-lactones was developed recently using the *Z*-selective cross-metathesis of (±)-MeJA and 3-butenyl acetate (both compounds are commercially available and inexpensive) resulting in the (±)-1-acetate derivative in excellent yield (>80%) and *Z*-selectivity (>90%) (Jimenez-Aleman et al., 2015b). Saponification of the (±)-1-acetate derivative (>85% yield) and conjugation to L-Ile resulted in the 1-hydroxy-12-L-Ile derivative. Finally, this derivative was exposed to macrolactonization resulting in enantiomerically pure macrolactones in only three steps. In agreement with the previous studies (Jimenez-Aleman et al., 2015a), these macrolactones also induced the accumulation of nicotine suggesting that these compounds open the possibility of uncoupling defence and growth in plants by using small molecules.

So far, JAs are only accessible today in industry through very tedious multistep synthesis (Chapuis, 2012). Together, the improvements introduced, including enantiodivergent routes that prevent the formation of all possible isomers (Nájera et al., 2020), and with total yields of around 33% are promising. However, further improvements of these multistep synthesis pathways are still necessary in order to increase the overall yield even from cheap starting materials.

## Fungi as Producers of JAs

The first report on JA production by microbes was published already 50 years ago (Broadbent, Hemming & Turner, 1968). These authors obtained JA from a culture of *L. theobromae* in a culture medium containing glucose, glycerol or a mixture of both as carbon source, as well as sodium nitrate, potassium nitrate or ammonium nitrate as nitrogen source. JA reached a concentration of 475 mg L^−1^ and a productivity of 37 mg L^−1^ d^−1^. In order to purify the produced JA, biomass was removed by filtration and the filtrate was acidified and further extracted with ethyl acetate. Three years later, JA biosynthesis was reported in a concentration of 500 mg L^−1^ and a productivity of 38 mg L^−1^ d^−1^ from *L. theobromae*, using a surface culture in 1 L ceramic vessels with Czapek medium (Aldridge et al., 1971). These authors also observed that the culture supernatant inhibited the growth of higher plants and that the active component was JA. Similar results were obtained by *L. theobromae* strain D7/2 isolated from orange and cacao residues (Miersch et al., 1987). This strain was grown in a liquid medium based on sucrose, soybean meal, corn steep liquor and salt solution with a JA concentration and productivity of 500 mg L^−1^ and 71 mg L^−1^ d^−1^, respectively.

The same authors performed a screening for JA production using 46 species of *Ascomycetes* and *Basiodimicetes* belonging to 23 different genera (*Agrocybe*, *Aspergillus*, *Collybia*, *Coprinus*, *Cunninghamella*, *Daedalea*, *Fomes*, *Fusarium*, *Gleooporus*, *Homoconis*, *Marasmius*, *Mucor*, *Mycena*, *Paecilomyces*, *Phellinus*, *Penicillium*, *Pleurotus*, *Polyporus*, *Rhizoctonia*, *Stropharia*, *Talaromyces*, *Trametes* and *Trichoderma*) that were grown under the same conditions as *L. theobromae*. In this screening trial, *Collibya*, *Coprinus* and *Mycena* were the best producers of JA. However, the JA concentrations were four to eight times lower than those found with *L. theobromae* cultures (Miersch et al., 1993). In addition, some mutants of *G. fujikuroi* were also able to produce JA in culture supernatants with a maximum amount of 2.5 mg L^−1^ (Miersch et al., 1992). Similarly, mycorrhizal fungi such as *Laccaria laccata* and *Pisolithus tinctorius* were identified as JA producers but again only in trace amounts (Miersch, Regvar & Wasternack, 1999).

A mutant approach was applied to obtain better JA producers of *L. theobromae* (Patel & Thakkar, 2015). The mutants were generated using ethylmethanesulfonate and two mutants were isolated having the capacity to produce JA with 70 mg L^−1^ and 78 mg L^−1^ compared to wild type 32 mg L^−1^.

However, the highest rates for JA production were described however for *Diplodia gossypina* strain ATCC 10936 (Farbood et al., 2001). Under optimal culture conditions, the JA concentration and productivity were 1,200 mg L^−1^ and 171 mg L^−1^ d^−1^, respectively. This study even included the up scaling of JA production up to a volume of 150 L. Therefore, the microorganisms that provide the highest potential for JA production are the ascomycete fungi from strains of *Diplodia* and *Lasiodiplodia* genera.

## Culture Conditions for JA Production

Although the annual demand for JA increases primarily for applications in perfume production and flavourings (Dhandhukia & Thakkar, 2007a), there are still only a few reports published related to the practical aspects of the commercial production of JA as shown in Table 2 and most of the information and strains is still only published in patents.

**Table 2:** Studies of jasmonic acid production by microbial way reported in the literature.

Microorganism	Experimental Procedure	Yield (mg L^−1^); Productivity (mg L^−1^ d^−1^)	References
*Lasiodiplodia theobromae* strain S22L	Surface culture, ceramic vessels (1L)	475; 37	Broadbent, Hemming & Turner (1968)
*L. theobromae*	Surface culture, ceramic vessels (1L)	500; 38	Aldridge et al. (1971)
*L. theobromae* strain D7/2	Surface culture (static), Erlenmeyer 400 mL	500; 71	Miersch et al. (1984, 1987)
*L. theobromae* strain 715	Surface culture (static), Erlenmeyer 250 mL	1,200; 120	Altuna et al. (1996)
*Lasiodiplodia* sp. strain 2334	Surface culture (static), Erlenmeyer 100 mL	900; 90	Eng, Gutiérrez-Rojas & Favela-Torres (1998)
*Fusarium oxysporum f* sp *matthiolae* strain 247.61	Surface culture (static), Bottle (2,000 mL)	0.5; 0.01	Miersch, Bohlmann & Wasternack, 1999
*L. theobromae* strain 715	Surface culture (static), Erlenmeyer 500 mL	1,000; 83	Almeida et al. (1999)
*Diplodia gossypina* strain ATCC 10936	Erlenmeyer 500 mL, agitation velocity 200 rpm	1,200; 171	Farbood et al. (2001)
	Reactor 150 L, agitation velocity 150 rpm	120; 17	
*D. gossypina* strain ATCC 10936	Erlenmeyer 500 mL, agitation velocity 200 rpm	600; 86	Inho, Kyoungju & Yonghwi (2006)
*L. theobromae* strain MTCC 3068	Surface culture (static), Erlenmeyer 250 mL	299; 43	Dhandhukia & Thakkar (2007a)
*L. theobromae* strain RC1	Surface culture (static), Erlenmeyer 250 mL	550; 56	Eng, Gutierrez-Rojas & Favela-Torres (2008)
*Lasiodiplodia* sp. strain 2334	Surface culture (static), Erlenmeyer 250 mL	1,270; 127	Eng (2012)
*Botryosphaeria rhodina*	Surface culture (static) Erlenmeyer 250 mL	352; 25	Dos Santos et al. (2014)
*L. theobromae*	Surface culture (static), Erlenmeyer 250 mL	784; 56	Laredo-Alcalá et al. (2016)
	Solid state fermentation, Erlenmeyer 125 mL	23 mg g^−1^; 2 mg g^−1^ d^−1^	
*L. theobromae* strain 2334	Surface culture (static), Erlenmeyer 500 mL	1,250; 139	Eng et al. (2016)
*F. oxysporum* f. sp. *tulipae*	Shaking culture (100rpm) Erlenmeyer 250–500 mL	230; 15	Oliw & Hamberg, 2017
*L. theobromae* strain 3C	Surface culture (static), Erlenmeyer 250 mL	565; 38	Laredo-Alcalá et al. (2016)

The ability of fungi to produce JA varies between strains from 1 mg L^−1^ to 1,300 mg L^−1^ of JA, even of the same species (Dhandhukia & Thakkar, 2007a; Eng, Gutiérrez-Rojas & Favela-Torres, 1998; Farbood et al., 2001). Therefore, the first strains of *L. theobromae* or *D. gossypina* were screened for JA production in order to select strains with higher productivity (Altuna et al., 1996; Eng, 2012; Farbood et al., 2001).

Batch fermentation in static conditions using a stationary Fernbach flask culture, an aseptic stationary tray culture or Erlenmeyers flasks were tested between 5 and 10 days at temperatures between 27 °C and 30 °C and slightly acidic initial pH values between 5 and 6 of the culture medium (Altuna et al., 1996; Farbood et al., 2001; Miersch et al., 1987). As a carbon source for producing JA, soybean meal, citrus pulp, corn steep liquor and milk serum were used and supplemented with oilseed meal, which can supply sources of protein, minerals and water soluble vitamins (Miersch et al., 1987). However, using more complex media had the drawback of needing more complicated processes for purifying JA for some applications such as in perfumery, a removal of malodorous compounds and allergens is required. Another drawback is that the composition thereof is not constant and therefore results may be difficult to reproduce. Primarily synthetic media were used that are based on sucrose or glucose as carbon source and mineral salts such as potassium nitrate as nitrogen source, with the addition of monobasic potassium phosphate, ammonium molybdate, and the sulfates of magnesium, iron, zinc and copper, respectively (Almeida et al., 1999; Miersch et al., 1987). Also only one type of carbon source (glucose or sucrose) can be used for JA production (Eng, Gutiérrez-Rojas & Favela-Torres, 1998). Then, catabolic repression is not evident as it occurs in other biosynthic routes of secondary metabolites in fungi. However, Farbood et al. used glucose or a mixture of glucose and xylose in their studies of strain selection and JA production with *D. gossypina* strain ATCC 10936 (Farbood et al., 2001). Broadbent, Hemming & Turner (1968) reported that sucrose, glucose, glycerol or mixtures of these carbon sources allowed a higher production of JA with *L. theobromae* strain S22L than the use of a single carbon source. Therefore, it is possible to use either a single or a mixed carbon source depending on the strain used.

The same was true in case of the nitrogen source when ammonium salts were replaced by nitrate salts (Eng, Gutiérrez-Rojas & Favela-Torres, 1998; Günther et al., 1989). The consumption of ammonium ions by the fungus during its growth very likely generates an acid pH in the culture medium, which could be responsible for the slow growth and therefore to the low JA production.

An early study showed that the addition of an inductor is not required to produce JA in synthetic culture medium (Miersch et al., 1987). In fact, the addition of 18:3(n-3) (1 µM) (Dos Santos et al., 2014) or edible oil (1 g L^−1^) (Eng, 1996) as substrate and fatty acid source for JA synthesis to the culture media of *Botryosphaeria rhodina* strain Kinf 3.1 or *Lasiodiplodia* sp. strain 2,334, respectively, was not significantly favoured in the JA production. However, the addition of yeast extract and/or soy peptone as a source of vitamins and cofactors to the culture medium stimulated the rate of JA biosynthesis (Dhandhukia & Thakkar, 2008; Eng, 2012; Eng, Gutierrez-Rojas & Favela-Torres, 2008; Farbood et al., 2001). In fact, the addition of these nutrients could cause a positive effect on the growth of these fungi and may promote an early onset of JA synthesis by decreasing the time at which the maximal production and stationary phase is reached.

Under these culture conditions JA production took place at the late exponential growth phase or stationary phase showing a behaviour similar to the accumulation of secondary metabolites (Eng et al., 2016) and may only partially be associated with the growth phase of the culture (Dhandhukia & Thakkar, 2007a). Using these optimized culture conditions JA production levels reached 500–1,300 mg L^−1^ and productivities of 28–170 mg L^−1^ d^−1^ (Table 2).

During static conditions, some *Lasiodiplodia* strains formed a mat on the surface of the culture medium (Eng, 1996). Therefore, the effect of the available surface area by increasing the vessel diameter may be another critical aspect for JA production. This was confirmed by a study on JA production by *L. theobromae* strain MTCC 3068 using the same amount of culture medium with Erlenmeyer flasks of 250, 500 and 1,000 mL in which the authors could show, that increasing the surface area of the culture lead to an increase of the JA yield (Dhandhukia & Thakkar, 2007a). In another study, the surface of the culture (100–500 mL) was simultaneously increased with the volume of the culture medium (25–100 mL). Here, JA production was highest at the largest surface area in combination with the highest volume of culture medium (Eng et al., 2016). However, an increase of the flask volume to 5 or even to 50 L and for the culture medium volume up to 10 L did not lead to further increases in JA yield (Eng, 2012), because the fungal mycelium grows on the surface of the medium. An increase in its volume limits only the diffusion of the nutrients from the culture medium to the fungal mycelium thereby limiting growth and JA production.

However, scaling up JA production in a fermenter or in a shaking incubator at 190 rpm and 30 °C with a dissolved oxygen saturation in the culture medium of up to 150 L (Farbood et al., 2001) as well as using a fixed inoculation ratio of 0.5 g L^−1^ of dry biomass of culture medium was shown to further improve JA yield (Miersch et al., 1987). In addition, it was of advantage to use homogenized mycelium and not spores (Almeida, Altuna & Michelena, 2001; Dos Santos et al., 2014; Dos Santos, Innocentini & Lourenco, 2014). Agitation turned out to be another critical aspect for JA production, because shaking speeds above 200 rpm lead to increased synthesis of extracellular polysaccharides that visibly increased the viscosity of the culture medium (Selbmann, Crognale & Petruccioli, 2004), which had a negative effect on JA production (Eng, Gutiérrez-Rojas & Favela-Torres, 1998; Miersch et al., 1987). These results suggest that stronger agitation of the culture lead to a higher concentration of dissolved oxygen in the medium and as a consequence, this microorganism produced more extracellular polysaccharides instead of JAs. In fact, Selbmann et al. confirmed these results by selecting *Botryosphaeria rhodina* DABAC-P82 for the production of exopolysaccharides using this strategy (Selbmann, Crognale & Petruccioli, 2004; Selbmann, Stingele & Petruccioli, 2003). This fungal strain was capable of producing up to 17.7 ± 0.8 g L^−1^ of an exopolysaccharide after only 24 h. The production of this exopolysaccharide was even further increased in stirred fermenters or with propeller turbines by increasing the stirring speed from 300 to 500 rpm. These extracellular polysaccharides may be used by these fungi to form capsules, which may protect them against the stress caused by the agitation of the culture medium.

JA was also obtained by solid-state fermentation (SSF) from *Lasiodiplodia* sp. strain 2334 using columns with sugar cane bagasse impregnated as support, at 30 °C and with a similar culture medium that was used in liquid fermentation. JA productivity was two times higher in SSF probably due to growth conditions that were more similar to the natural environment of this fungus (Eng, 1996) and monosaccharides released by the fungal cellulolytic activity on the substrate in the stationary late phase. Using similar conditions, JA productivity of a strain of *B. theobromae* isolated from cacao tissue was reported to be three times higher by SSF as with submerged fermentation (Laredo-Alcalá et al., 2016). Therefore, the most promising approach is to continue studying this fermentation method taking into account the benefits of SSF with low costs and due to the absence of free water, small fermenters can be used and therefore less effort is required for the separation processes. The main drawback so far is its control and dissipating the metabolic heat produced in the reactors, which potentially could reduce the fungal activity.

Finally, it should be noted that JA production was possible with *D. gossypina* strain ATCC 10936 in stirred fermenters of 150 L at an agitation velocity of 450 rpm, but productivity decreased at about two times with respect to the production in 500 mL Erlenmeyer flasks agitated at a speed of 200 rpm (Farbood et al., 2001).

The progress made in the production of JA by fungi is undoubted. However, the development of new studies starting with the selection and mutagenesis of the producer strains, culture media and conditions to scale up production, the biosynthesis pathway and the genes involved and the evaluation of effectiveness of their applications are essential and should continue to be explored.

The broader use of JAs has been limited by the high costs of commercial production so far. The production of JAs from fungi, via fermentation, has emerged as a promising alternative to reduce production costs. The use of simple and relatively inexpensive culture conditions is an attractive strategy to bring JA production with these strains to industrial levels. It may allow to significantly reduce the production costs which makes the development of future bioproducts more attractive.

## Patents

There is a growing number of patents describing the production and application of JAs since the 60’s and their quantity have increased during the last decades showing a growing interest in this substance class (Pirbalouti, Sajjadi & Parang, 2014). In the beginning patents dealing with the isolation, detection and culture conditions for production of JAs in microorganisms such as *L. theobromae* were published (Aldridge et al., 1971; Broadbent, Hemming & Turner, 1968; Farbood et al., 2001; Günther et al., 1989; Miersch et al., 1984; Yoshihara, 2007).

Other topics deal with agricultural applications of JAs in order to improve plant yield most likely by inducing plants defence against herbivores and pathogens, including the cultivation of algae and edible fungi (Beibei et al., 2019; Beibei et al., 2018; Dathe et al., 1990; Guo et al., 2019; Ryan & Farmer, 1991; Wang et al., 2020b; Zhang et al., 2020). Recently these effects were combined with new formulations for JAs in water in combination with herbicides, pesticides, bioactive or biological seed treatments and semiochemicals (Deng, Lan & Zhao, 2020; Lulai, Orr & Glynn, 1995; Marks, 2012; Pirbalouti, Sajjadi & Parang, 2014; Wang et al., 2020a, 2020c). The application of MeJA to grapes in order to improve the quality of machine-harvested raisin grapes allowed the harvest without damaging the fruit or plants associated with traditional mechanical harvesting and thereby eliminating the need for expensive hand picking (Pirbalouti, Sajjadi & Parang, 2014). Meanwhile a method was also reported for improving the turf grass quality (Mcelroy, 2011) and others to applicate in plant leaf blade flavonoids that permit the accumulation of terpene lactones using MeJA (Wang, Guo & Feng, 2019). There is an only one patent claiming the use of a JA extract to inhibit the growth of the bacterium *Leuconostoc* sp. and thereby dextran production during the juice processing of the sugar cane industry (Michelena et al., 2010).

Nowadays, patents about JAs have expanded to medicinal, cosmetic and flavouring applications (Pirbalouti, Sajjadi & Parang, 2014). During the last 20 years, the vast majority of studies and inventions claim that JA, MeJA and dihydro-MeJA have anticancer activity against various forms of cancer (Fleischer & Fingrut, 2007; Fleischer, Herzberg & Kashman, 2012; Fleischer et al., 2010; Guzel et al., 2019; Herzberg, Harel & Mang, 2006; Martinez et al., 2010; Pirbalouti, Sajjadi & Parang, 2014). However, convincing evidence that supports these claims is still missing. Additional patents focus on improving the convenience and safety of their administration and on expanding the applications for the treatment, for example, the use of nanocarriers in order to increase the solubility of JAs, because these compounds are poorly water-soluble, not allowing an application by an intravenous route without an efficient nanostructured carrier system. A major problem is still that they are not easily delivered to cancerous cells, because they often degraded before they reach the tumour cells (Da Silva et al., 2014; Katona, Vempati & Savir, 2015; Lopes, 2014). JAs are also used as skin care and hair care products, for example, for treating hair, the scalp, dry and greasy skin (Bababunmi, 2005; Broady, 2012; Dalko, 2006; Deyong, Mengjiao & Naixing, 2019; Malik, 2006; Pirbalouti, Sajjadi & Parang, 2014) and also in Bladder dysfunction (Pirbalouti, Sajjadi & Parang, 2014). Jasmone, MeJA, CJ and γ-jasmolactone are considered as the main odorous substances in the essential oil of jasmine flowers (jasmine oil) used in perfumes (Smets et al., 2020; Steinegger & Hansel, 1988). Other authors described the use of dihydro-MeJA as enhancer or imparter fragrances in or to a perfume composition, perfumed articles and colognes (Boden, Fujioka & Hanna, 1993). In addition, JAs are also used to flavour fruit beverages, confectionery like sweets and candy, cigarette, food products like cocoa and tooth cleansing products like toothpaste (Hurst et al., 2015; Hurst et al., 2011; Johnson, Paul & Favara, 1977; Mookherjee et al., 1981).

## Conclusions

Beside plants, fungi are additional producers of JAs, and those providing the highest yields for JA production are *Ascomycetes* from the genus *Lasiodiplodia* and *Diplodia*.

There is a great of diversity of JAs that are produced by fungi, but JA, MeJA and dihydro-MeJA have studied the most because of their numerous applications. In the fungus *L. theobromae*, plant-type jasmonate derivatives such as hydroxy and amino acids conjugates, as methyl and sulfate ester occur. In addition, derivatives being specific for fungi such as hydroxy-lactones, didehydro or dihomo-JAs are found. However, until today the function of JAs being produced by these fungi is not known. However, it can be assumed that they are involved regulating the interaction between plants and microorganisms.

Strategies to produce JAs via microbial chemical synthesis suffer still from low yields. In case of fungal production strategies, a the number of promising strains from the genus *Lasiodiplodia* and *Diplodia* have been selected, but they suffer from producing jasmonate mixtures and strategies for purifying the elaborated product are needed to develop an industrial processes for JA production.

The knowledge gained so far provides a promising basis for additional research on the interaction of these fungi with plants, the chemical nature of JA biosynthesis in fungi, mechanisms that regulate this pathway in fungi and design simpler and viable technological strategies to produce JAs in these fungi in order to satisfy the high demand for these products will be the next challenges in this field of research.

It is very likely that applications for JAs continue to increase in the biomedical, cosmetic, food and in agricultural sector, as soon as a better understanding of their biosynthesis and mode of action and their molecular interactions with biological targets will became available.

## Abbreviations

12-HSO4-JA: 12-hydroxy jasmonic acid sulfate
16:3 (=16:3(n-3)): raughanic acid
18:2 (=18:2(n-6)): linoleic acid, hexadecatrienoic acid
18:3 (=18:3(n-3)): α-linolenic acid
ABA: abscisic acid
AOC: allene oxide cyclase
AOS: allene oxide synthase
CA: curcurbic acid
MeCA: methyl curcurbate
CJ: cis-jasmone
ddh-JA: didehydro jasmonic acid
dn-OPDA: dinor-12-oxo-phytodienoic acid
GA3: gibberellic acid
JA: jasmonic acid
JAs: Jasmonates
JA-Leu: jasmonoyl leucine
JMT: JA carboxyl methyltransferase
MeJA: methyl jasmonate
LOX: lipoxygenase
OPC-4: 3-oxo-2(2′-pentenyl)-cyclopentane-1-butanoic acid
OPC-6: 3-oxo-2(2′-pentenyl)-cyclopentane-1-hexanoic acid
OPC-8: 10,11-dihydro-12-oxo-phytodienoic acid
OPDA: 12-oxo-phytodienoic acid
SSF: solid state fermentation
TA: tuberonic acid

## Additional Information and Declarations

### Data Availability

The following information was supplied regarding data availability: The research in this article did not generate any data or code; this is a literature review.
